# High Performance of Titanium Dioxide Reinforced Acrylonitrile Butadiene Rubber Composites

**DOI:** 10.3390/polym14235267

**Published:** 2022-12-02

**Authors:** Wannarat Chueangchayaphan, Piyawadee Luangchuang, Narong Chueangchayaphan

**Affiliations:** Faculty of Science and Industrial Technology, Prince of Songkla University, Surat Thani Campus, Surat Thani 84000, Thailand

**Keywords:** acrylonitrile butadiene rubber, titanium dioxide, mechanical properties, dielectric properties

## Abstract

Recently, dielectric elastomer actuators (DEA) have emerged as one of the most promising materials for use in soft robots. However, DEA needs a high operating voltage and high mechanical properties. By increasing the dielectric constant of elastomeric materials, it is possible to decrease the operating voltage required. Thus, elastomeric composites with a high dielectric constant and strong mechanical properties are of interest. The aim of this research was to investigate the effect of titanium dioxide (TiO_2_) content ranging from 0 to 110 phr on the cure characteristics, and physical, dielectric, dynamic mechanical, and morphological properties of acrylonitrile butadiene rubber (NBR) composites. The addition of TiO_2_ reduced the scorch time (*t_s_*_1_) as well as the optimum cure time (*t*_*c*90_) but increased the cure rate index (CRI), minimum torque (*M_L_*), maximum torque (*M_H_*), and delta torque (*M_H_* − *M_L_*). The optimal TiO_2_ content for maximum tensile strength and elongation at break was 90 phr. Tensile strength and elongation at break were increased by 144.8% and 40.1%, respectively, over pure NBR. A significant mechanical property improvement was observed for TiO_2_-filled composites due to the good dispersion of TiO_2_ in the NBR matrix, which was confirmed by scanning electron microscopy (SEM). Moreover, incorporating TiO_2_ filler gave a higher storage modulus, a shift in glass transition temperature (T_g_) to a higher temperature, and reduced damping in dynamic mechanical thermal analysis (DMTA). The addition of TiO_2_ to NBR rubber increased the dielectric constant of the resultant composites in the tested frequency range from 10^2^ to 10^5^ Hz. As a result, TiO_2_-filled NBR composite has a high potential for dielectric elastomer actuator applications.

## 1. Introduction

Dielectric elastomer actuators (DEA) are a class of electroactive polymers that deform when an electric field is applied. It can exhibit large deformation, fast response, and high energy density under the external electrical field. DEA can be used in several applications, including electric-induced actuators, soft robotic structures, and smart electronic skins [1,2]. In order to induce deformation, DEA requires a high operating voltage (several kilovolts), which limits its commercial viability. However, the required operating voltage can be reduced by increasing the energy density of the elastomer, which can be achieved by decreasing the thickness of the dielectric elastomer or increasing the dielectric constant of elastomeric materials [1,3]. A conventional method for enhancing the dielectric constant of an elastomer involves inserting dipole groups into the rubber chain or incorporating a filler with a high dielectric constant [3,4].

Acrylonitrile butadiene rubber (NBR) is a copolymer of acrylonitrile and butadiene monomers. Due to acrylonitrile having a functional group (C≡N), NBR presents a specialty polar rubber with a high dielectric constant. NBR has been extensively used as a workhorse in industrial and automotive rubber products [5] because it has beneficial properties such as a broad temperature application range, bonding performance, excellent oil and solvent resistance, and relatively low cost [6,7]. However, NBR does not exhibit self-reinforcing behaviors [8], as there is no strain-induced crystallization due to its irregular molecular structure of random copolymers, resulting in comparatively weak mechanical properties [9]. Thus, efforts have to be directed at finding an appropriate filler for NBR to achieve high-performance products with good mechanical properties. Several fillers, namely clay [10,11,12], carbon black [13,14,15,16], carbon nanotubes [16,17,18], and silica [16,19,20,21]; have been tested for NBR.

Fillers aim to reduce cost and improve mechanical, thermal, and electrical properties. Titanium dioxide (TiO_2_) has been attractive because of its great characteristics, such as safe production, non-toxicity, high reactivity, chemical stability, electrochemical properties, biocompatibility, and low cost [22,23]. It is widely employed as a white pigment in plastics, paints, paper, and cosmetics [22], as well as a UV absorber, and antibacterial agent, in functional devices [24], semiconductors, and ceramics [25]. In addition, TiO_2_ has been further used in glass, polyvinyl alcohol (PVA), and polycarbonate such as gamma-ray shielding [26], alkaline anion exchange membranes [27] and improved in environmental stress cracking [28], respectively. Furthermore, TiO_2_ has been used as a high dielectric constant filler (approximately 100). In the last decade, the incorporation of TiO_2_ into polymers to improve physical, thermal, mechanical, and dielectric properties has been explored. For example, Yang and Kofinas 2007 studied nano-TiO_2_-filled sulfonated styrene-b-(ethylene-ran-butylene)-b-styrene (S-SEBS) block copolymers by using vinyltrimethoxysilane as a crosslinker in order to decrease the dielectric loss from the free sulfonic acid groups [29]. Zhang et al. reported that rod-shaped TiO_2_ nanoparticles act as effective fillers for improving the thermal stability and impact strength of rigid poly(vinyl chloride) (PVC) nanocomposites [30]. Qi et al. used surface-modified rutile-type titanium dioxide (CST) nanorods as a UV absorber in polypropylene (PP) [31], while Nasrin et al. investigated TiO_2_-filled isotactic polypropylene (iPP) composites with various TiO_2_ contents. The dielectric constant decreased and the number of voids and holes on the composite surfaces increased with TiO_2_ concentration, due to the incompatibility between TiO_2_ and iPP matrix [32]. Madidi et al. demonstrated that the introduction of 5 wt.% nano-TiO_2_ and 10 wt.% micro-TiO_2_ improved the dielectric properties of silicone rubber/TiO_2_ composites. Additionally, an increased TiO_2_ concentration increased the relative permittivity of the composite [33]. Commonly, TiO_2_ has been utilized as an inexpensive inorganic pigment in rubber industries [34]. Nevertheless, the introduction of TiO_2_ in the NBR for dielectric and mechanical properties via melt mixing is still largely unexplored. Some literature reports on polydopamine (PDA) coated nano-TiO_2_ particles varying from 0 to 30 phr for improved TiO_2_ dispersion in NBR by using peroxide cure system [35], but this showed an increase in tensile strength of the composites from 1.757 MPa for pure NBR to 2.287 for 30 phr TiO_2_-PDA/NBR. Peroxide-cured rubbers exhibit good aging resistance and low compression set at high temperatures, but the disadvantages include low scorch safety, tensile strength, tear strength, and worse dynamic and elastic properties compared to sulfur curing system [8,35]. This subsequently leads to the incorporation of TiO_2_ microparticles within the NBR matrix using a sulfur curing system to improve the mechanical properties of composites.

In the present work, there is a great deal of interest in elastomeric composites with a high dielectric constant and robust mechanical properties. NBR composites were prepared by using micro-TiO_2_ as reinforcing filler without any compatibilizer or coupling agent. The main purpose of this study was to investigate the influences of high TiO_2_ loading varying from 0 to 110 phr on cure characteristics, and mechanical, and dielectric properties of the filled NBR composites.

## 2. Materials and Methods

### 2.1. Materials and Sample Preparation

Table 1 exhibits the materials, their suppliers, and formulation. Acrylonitrile butadiene rubber (NBR) with an acrylonitrile content of 33% was used in this research. Titanium dioxide (TiO_2_) was analytical reagent grade with 99.5% purity, the particle size is 21.69 ± 10.33 μm. All materials were used as received without any purification. Composites were blended in a 500 mL internal mixer at 60 °C with a rotor speed of 60 rpm. Mixing time and the sequence of adding ingredients were the same for all the composites, as listed in Table 1. Rubber compounds were sheeted out by using a two-roll mill and were kept at room temperature for 24 h before cure evaluation with a moving die rheometer (MDR) at 160 °C according to ASTM2240. The minimum torque (*M_L_*) and maximum torque (*M_H_*), torque difference (*M_H_* − *M_L_*), scorch time (*t_s_*_1_), and cure time (*t_c_*_90_) were acquired. Additionally, 150 mm × 160 mm × 2 mm rubber composite sheets were obtained from compression molding at 160 °C for duration in accordance with their respective *t_c_*_90_. Mooney viscosity (*M_L_* (1 + 4), 100 °C) was determined at 100 °C with the large rotor for 4 min after preheating for 1 min according to ASTM D1646.

### 2.2. Characterization and Testing

A universal tensile test machine (Tinius Olsen, model 10ST, Salfords, England) was utilized to perform mechanical testing on rubber composites in accordance with ASTM D-412 at room temperature with a fixed extension speed of 500 mm/min at 23 ± 2 °C. The moduli at 100% and 300% elongation, as well as the tensile strength and elongation at break, are provided. The hardness of the NBR/TiO_2_ composites was tested using a Shore A durometer (Montech Rubber Testing Solutions, Hardness HT3000, Buchen, Germany)) using 6 mm thick samples in accordance with ASTM D2240. Dynamic mechanical thermal analysis (DMTA 8000, PerkinElmer Inc., Waltham, MA, United States) was used to characterize the thermal analysis of NBR/TiO_2_ composites. The dynamic mechanical thermal experiment was carried out in tension mode at a heating rate of 3 °C/min and a strain of 0.1%. Temperature scanning and frequency scanning were carried out between −50 °C and 50 °C as well as 0.1 Hz and 100 Hz, respectively. At 25 °C, the dielectric constant of the vulcanizates was measured with an impedance analyzer (Agilent Technologies, Agilent 4285A, Santa Clara, CA, USA) across the frequency ranges of 10^2^–10^5^ Hz of AC at 1 V. The samples were placed between two parallel plates of the 5 mm diameter electrodes. SEM (FEI Quanta 400, Jeol Ltd., Tokyo, Japan) was utilized to detect TiO_2_ dispersion in an NBR matrix. Cryogenic fracturing in liquid nitrogen was used to create a new cross-sectional surface for the samples. The fractured specimens were then sputtered with a thin layer of gold under a vacuum before being imagined.

## 3. Results and Discussion

### 3.1. Cure Characteristics of NBR/TiO_2_ Composites

**Figure 1:**
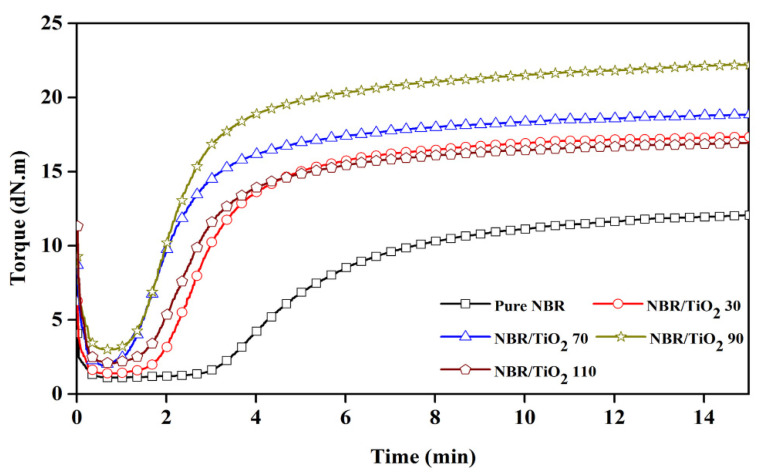
Cure curves of TiO_2_-filled NBR composites with various TiO_2_ loadings.

Cure characteristics of the NBR composites with varied TiO_2_ content are demonstrated and listed in Figure 1 and Table 2, respectively. The incorporation of TiO_2_ caused a shortened scorch time (*t_s_*_1_) and cure time (*t_c_*_90_). Increased TiO_2_ content reduced *t*_*s*1_ and *t*_*c*90_ because TiO_2_ metal oxide acts as a co-activator stimulating the crosslinking during sulfur curing. This is supported by the cure rate index (CRI) used to evaluate cure kinetics. The CRI increased with TiO_2_ contents up to 30 phr and then remained constant. This supports the TiO_2_ activating faster curing in the TiO_2_-filled NBR composites compared to the unfilled compound.

**Figure 2:**
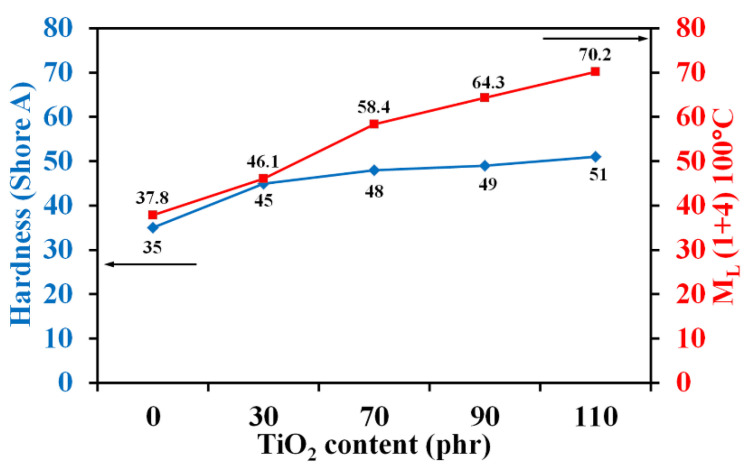
Mooney viscosity and hardness of TiO_2_-filled NBR composites with various TiO_2_ loadings.

The viscosity of the compound and stiffness of the vulcanizes are indirectly reflected in the minimum torque (*M_L_*) and the maximum torque (*M_H_*), respectively. With increasing TiO_2_ content, *M_L_* displayed an increasing trend because TiO_2_ particles obstructed molecular mobility with increased friction and high resistance to flow. However, *M_L_* tended to decrease at 110 phr of TiO_2_ content as a result of filler agglomeration. Figure 2 shows the correlation between the hardness of NBR filled with TiO_2_ and viscosity. It is seen that TiO_2_-filled NBR composites exhibited much higher viscosities than virgin NBR due to the hydrodynamic effects of filler, filler–filler interactions, and filler–rubber interactions [36]. Moreover, the Mooney viscosities of TiO_2_-filled NBR composites increased from 37.8 to 70.2 MU with TiO_2_ loading, since an increased number of solid particles in the soft matrix caused more movement restrictions [37].

**Scheme 1:**
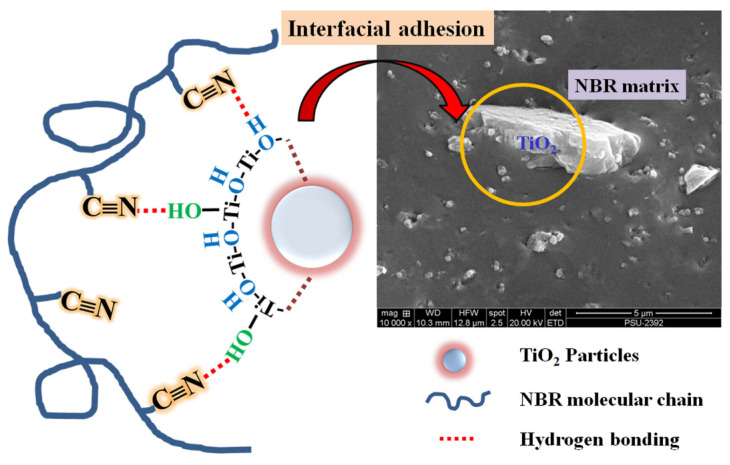
Schematic diagram illustrating physical bonding and interfacial adhesion in TiO_2_-filled NBR composites.

The torque difference (*M_H_ − M_L_*) indirectly reflects the crosslink density in rubber vulcanizates. *M_H_−M_L_* increased with the addition of TiO_2_ indicating a higher crosslink density caused by TiO_2_ due to strong filler–rubber interactions: the physical interactions between polar -C≡N groups in NBR and –OH groups on TiO_2_ surfaces. These surface hydroxyl groups, both of terminal OH and bridge OH, were generated by the reaction of surface oxide with moisture in the air [38,39]. The proposed physical interaction in TiO_2_-filled NBR composites is shown in Scheme 1.

### 3.2. Mechanical Properties

**Figure 3:**
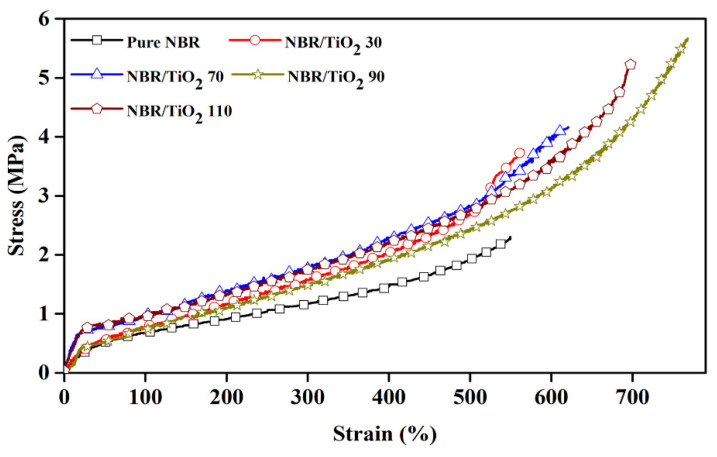
Stress–strain behavior of TiO_2_-filled NBR composites with various TiO_2_ loadings.

**Scheme 2:**
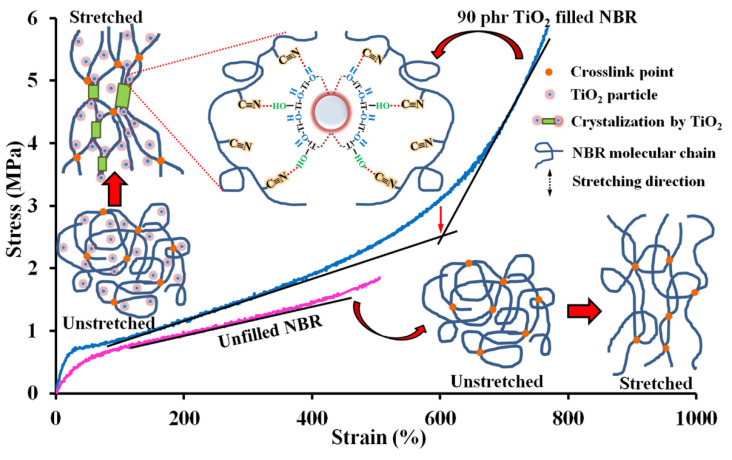
Schematic illustration of the proposed reinforcing mechanism and strain-induced crystallization in TiO_2_-filled NBR composites.

Figure 3 displays the stress–strain curves of the NBR composites. Pure NBR exhibited the lowest stress–strain curve with elongation at break ~550%, and without self-reinforcing behavior due to the irregular random copolymer structure, leading to insufficient mechanical properties [9]. It was found that the stress steadily increased with applied strain and then escalated sharply for TiO_2_-filled NBR composites. The abrupt increase in stress was observed at a high strain (>500% strain), especially with 90 phr TiO_2_ content, as opposed to the unfilled NBR composite that did not show strain-induced crystallization. The strain at the onset of the stress upturn corresponded to the onset of crystallization [40]. In the presence of TiO_2_, stronger chain alignment in the strain direction was caused by the additional crosslinks created by filler–rubber interactions [41], as seen in Scheme 2. This corroborates that the NBR molecular chain orientation was assisted by TiO_2_ particles, to perform interfacial crystallization during stretching. Moreover, TiO_2_-filled NBR composites showed an increased slope of the stress–strain curve compared to pure NBR, indicating stiffening. This matches the responses in *M_H_* and hardness discussed earlier.

Table 3 lists the mechanical properties of the various NBR composites. Most mechanical properties, namely tensile strength, elongation at break, moduli at 100% and 300% elongations, and hardness, increased with TiO_2_ loading indicating that this acted as a reinforcing filler in the NBR matrix. Tensile strength and elongation at break improved with increasing TiO_2_ content from 0 phr to a maximum at 90 phr. The 90 phr TiO_2_ content showed a 144.8% increase in tensile strength and a 40.1% increase in elongation at break. This was primarily attributed to the good filler-rubber interactions leading to a good filler dispersion in the NBR matrix and efficient stress transfer from matrix to filler [42]. A good ability in a composite to transfer applied stress relies on strong filler–matrix interactions. Secondarily, the compatibility of –OH functional groups in TiO_2_ and -C≡N can create strong filler–matrix interfacial adhesion (Scheme 1). Consequently, the filler–filler bonds and filler–rubber bonds in TiO_2_-filled NBR composites contributed to the total crosslinking [43]. On further increasing TiO_2_ content up to 110 phr, the tensile strength and elongation at break slightly decreased, probably due to agglomeration of TiO_2_ in the NBR matrix and thereby degraded filler–rubber interactions [44], while the matrix phase continuity was also impeded. Stress transfer was obstructed and weaker composites with lower tensile strength, lesser flexibility, and higher stiffness were obtained [45].

Moduli at 100% and 300% elongations are indirectly correlated to stiffness and rigidity. Table 3 shows that the moduli at 100% and 300% elongations were higher for TiO_2_-filled composites than for unfilled vulcanizate. The moduli at 100% and 300% elongations were increased by 111% and 139%, respectively, for NBR composites with 110 phr TiO_2_ content. This indicates that the stiffness of the composites had increased due to crosslinking, in agreement with *M_H_* and *M_H_−M_L_* results previously discussed with the cure characteristics. Considering the reinforcing index, the ratio of the modulus at 300% elongation to modulus at 100% elongation (M300/M100) [36], was higher for TiO_2_-filled NBR composites than for pure NBR vulcanizate. This was probably due to good interactions between NBR rubber and TiO_2_ filler. This is confirming that TiO_2_ acted as a reinforcing filler in NBR rubber. The 70 phr TiO_2_-filled composites exhibited the highest reinforcement index. On further increasing TiO_2_ content, the reinforcement index tended to decrease due to filler agglomeration.

The resistance to surface penetration by a material is evaluated by its hardness. It was found that the hardness of NBR composites is affected by TiO_2_ content as seen in Table 3. The NBR composite reinforced with 110 phr TiO_2_ loading exhibited an increase of approximately 46% from the hardness of the pure NBR vulcanizate. This is explained by the increased crosslink density in NBR composites, related to the higher torque difference seen earlier, as well as the rigid inorganic TiO_2_ particles being noticeably stiffer than the rubber matrix [46]. As more TiO_2_ particles were introduced into the NBR matrix, the elasticity diminished [47]. Chokanandsombat and Sirisinha explained an increase in hardness with a dynamic effect in which the rubber matrix was diluted by rigid metal oxide fillers [48]. Moreover, an increase in the TiO_2_ loading of the NBR matrix reduced the free volume because of the filling of the space by complex formations of Ti^3+^ and O^−^ ions [27], leading to increased resistance to indentation for the composite.

### 3.3. Morphology Characterization

**Figure 4:**
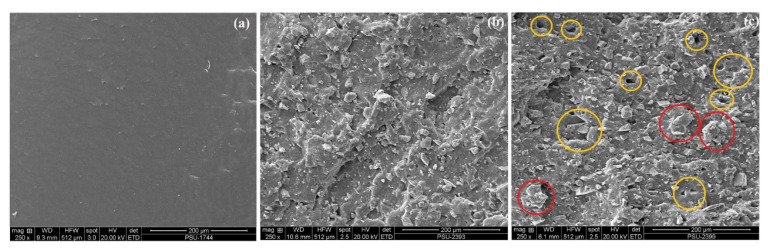
SEM images of fracture surfaces on TiO_2_-filled NBR composites with (**a**) 0 phr TiO_2_ (or pure NBR), (**b**) 70 phr TiO_2_, and (**c**) 110 phr TiO_2_.

SEM was used to determine the compatibility and dispersion of filler in the rubber matrix which affected the properties of the filled composites. Figure 4 exhibits SEM micrographs taken from fracture surfaces of NBR composites. Smooth surfaces are seen in Figure 4a due to the lack of an inorganic filler in the NBR matrix. SEM images support the prior tensile properties. This shows that incorporating TiO_2_ in the NBR matrix induced rougher fracture surfaces so more energy was needed to break the sample. The strong hydrogen bonds between TiO_2_ particles and the NBR matrix can prevent the formation of defects that initiate or propagate fractures. This matches the improved mechanical properties TiO_2_-filled composites compared to pure NBR vulcanizate. Good dispersion and compatibility (Scheme 2) between TiO_2_ and NBR played major roles in improved stress transfer between the NBR rubber matrix and TiO_2_ filler. However, some cavities (yellow circle) and TiO_2_ aggregates (red circle) in the NBR matrix were evidenced when TiO_2_ loading exceeded 90 phr (Figure 4c) so less energy was needed for failure.

### 3.4. Dynamic Mechanical Analysis

Dynamic mechanical properties inform about storage modulus, loss modulus, and loss tangent (tan δ). The storage modulus relates to the stiffness, while the loss modulus is associated with viscous response. Tan δ is the ratio of loss modulus to storage modulus, relating to the molecular mobility and phase transition [49].

**Figure 5:**
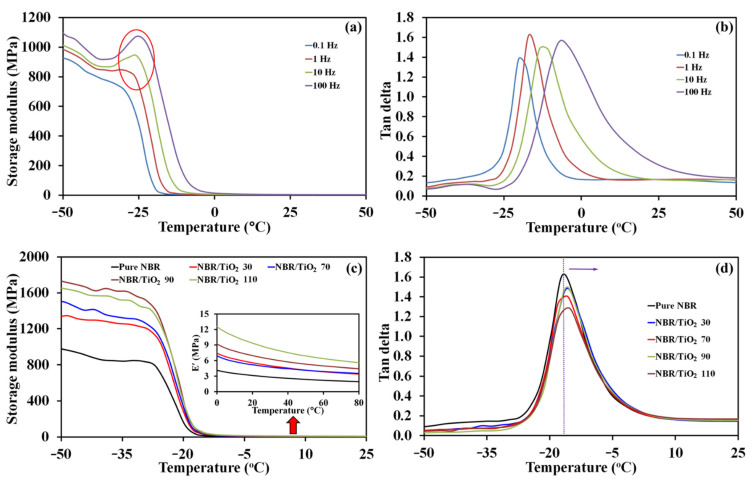
(**a**) Storage modulus, and (**b**) tan δ as functions of temperature for pure NBR at several frequencies; (**c**) storage modulus, and (**d**) tan δ as functions of temperature for TiO_2_-filled NBR composites with various TiO_2_ loadings at 1 Hz.

Figure 5a,b show the typical variation with temperature in storage modulus and tan δ, respectively, at the frequencies 0.1, 1, 10 and 100 Hz for pure NBR. Since rubber is a viscoelastic material, its properties display time dependence where a high frequency translates into a short time. It was found that the storage modulus increased and shifted to higher temperature with increasing frequency, as seen in Figure 5a. This was because the rubber chains have little time to transition during high-frequency cyclic deformation, resulting in the material behaving more like an elastic solid with a larger storage modulus [50]. In addition, at a higher frequency, a peak or hump (red circle in Figure 5a) was observed in the storage modulus because molecular rearrangement eased the stress that had been trapped at temperatures below the glass transition temperature (T_g_) [51]. The dynamic mechanical thermal analysis represents an alternative method for measuring the glass transition temperature, which is the identified location of the tan δ peak. At T_g_, the expanding volume of the material with increasing temperature becomes sufficient to allow chain flow. It is clear that T_g_ increased with frequency from −20.3 °C to −4.1 °C, as shown in Figure 5b, while the intensity of tan δ peak tended to increase due to the rubber composite behaving more like an elastic solid at a higher frequency of deformation [50].

In addition, dynamic mechanical properties are used to assess the interactions between the inorganic TiO_2_-filler particles and the NBR matrix. Temperature dependence of the storage modulus (E′) and of tan δ are shown in Figure 5c,d, respectively. Figure 5c demonstrates that typical behavior of all storage modulus curves for vulcanized rubber, with three distinct states: the glassy high modulus state at very low temperature where molecule movements are tightly compressed; the transition state where the storage modulus sharply declines related to the coordinated chain segments in the amorphous phase simultaneously moving; and the rubbery plateau with modulus correlated to crosslink density or chain length between entanglements [51,52]. Increasing TiO_2_ content in the NBR matrix increased E′ in both the glassy and the rubbery regions. This was attributed to the stiffening effects of TiO_2_ rigid filler along with its interactions with the NBR matrix. In Figure 5d, the peak position of the tan delta is plotted, identifying T_g_ affected by the mobility of molecular segments. Relatively low tan δ is observed both below and above T_g_. This is because the molecular chains are immobilized and restricted in the glassy state, while the molecular chains can move more freely in the rubbery state. The slight shift in the T_g_ to higher temperatures with TiO_2_ loading was due to the good filler–rubber interactions limiting chain mobility, resulting in higher energy required for mobility. In addition, pure NBR has the highest tan δ at ~1.62 due to its flexible elastomer nature. Tan δ tends to decrease with TiO_2_ loading because of filler–rubber interactions via hydrogen bonds acting as physical crosslinks, obstructing chain mobility in TiO_2_-filled composites, and causing a less effective response to cyclic loading. The shift of T_g_ toward higher temperatures and decrease in tan δ support good compatibility between TiO_2_ filler and NBR rubber matrix.

### 3.5. Dielectric Properties of NBR/TiO_2_ Composites

**Figure 6:**
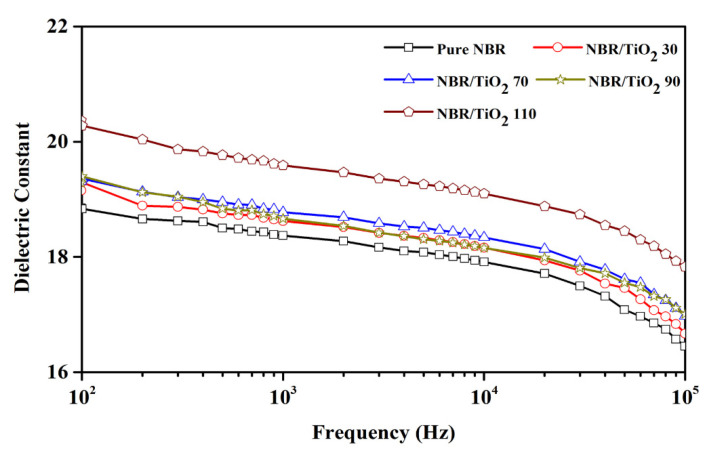
Dielectric constant of TiO_2_-filled NBR composites with various TiO_2_ loadings.

Figure 6 displays the dielectric constant versus frequency for different TiO_2_ loadings in NBR composites. There is a noticeable reduction in dielectric constant with frequency from 10^2^ to 10^5^ Hz in all cases because the electrical polarization is harder to follow at a higher frequency [53]. The maximum (~18.83) for pure NBR was observed at 10^2^ Hz because of strong contributions from interfacial and dipolar polarization in the low-frequency range [54]. The high dielectric constant of pure NBR was due to the orientation polarization of the -C≡N dipoles. On increasing the frequency to 10^5^ Hz, the dielectric constant decreased. This might be because the dipoles are incapable of reorienting rapidly enough to follow the applied frequency. With the introduction of TiO_2_, the dielectric constant of TiO_2_-filled composites increased with increasing TiO_2_ content because TiO_2_ has a higher dielectric constant than pure NBR. The 110 phr TiO_2_-filled composites showed the highest dielectric constant of 20.28 at a frequency of 10^2^ Hz. Furthermore, the increased dielectric constant was partly attributed to better dipole orientation polarization due to the segment arrangement of rubber chains [55]. After the incorporation of TiO_2_ filler, the composites had more interfaces enabling interfacial polarization [56].

## 4. Conclusions

TiO_2_ was incorporated as filler into NBR with loadings up to 110 phr. It has good compatibility and disperses efficiently in the NBR matrix due to the strong filler-rubber interactions via hydrogen bonds between hydroxyl on the TiO_2_ surface and acrylonitrile-functional groups, resulting in an increased cure rate index and crosslink density in TiO2-filled compounds. Accordingly, improved tensile strength, elongation at break, moduli at 100% and 300% elongations, and hardness were obtained. The 90 phr TiO_2_ content resulted in a 144.8% increase in tensile strength and a 40.1% increase in elongation at break. This was primarily due to good filler–rubber interactions, which resulted in good filler dispersion in the NBR matrix and efficient stress transfer from matrix to filler. The addition of TiO_2_ resulted in an increase in storage modulus, a slight shift in T_g_ upward, and less mechanical damping. The presence of TiO_2_ in the composites increased the dielectric constant of filled NBR. The 110 phr TiO_2_-filled composites had the highest dielectric constant of 20.28 at 10^2^ Hz. High dielectric constant and strong mechanical properties are two benefits of the TiO_2_-filled composites that were obtained. As a result, it could be implemented in dielectric elastomer actuators application.

## Figures and Tables

**Table 1:** Details of the materials and compounding formulations of TiO_2_-filled NBR composites.

Ingredient	Supplier	phr	Internal Mixer	Time (min)
NBR	Nantex public Co., Ltd. (Kaohsiung, Taiwan)	100	- Mastication of NBR	0
Sulphur	Vessel chemical public Co., Ltd. (Bangkok, Thailand)	1.5	- Addition of Sulphur	2
Zinc oxide	Bossoftical public Co., Ltd. (Songkla, Thailand)	5	- Addition of Zinc oxide	4
Stearic acid	Bossoftical public Co., Ltd. (Songkla, Thailand)	2	- Addition of stearic acid	6
MBTS	Vessel chemical public Co., Ltd. (Bangkok, Thailand)	1.5	- Addition of TiO_2_	7
TiO_2_	Labchem public Co., Ltd. (Surat Thani, Thailand)	0–110	- Addition of MBTS	10
			- Dumping	12

**Table 2:** Cure characteristics and Mooney viscosity of TiO_2_-filled NBR composites with various TiO_2_ loadings.

Sample	*M_L_*	*M_H_*	*M_H_* − *M_L_*	*t_s_*_1_	*t_c_*_90_	CRI	*M_L_* (1 + 4) 100 °C (MU)
	(dN.m)	(dN.m)	(dN.m)	(min)	(min)	(min^−1^)	
Pure NBR	1.06	12.15	11.09	3.17	9.68	15.35	37.80
NBR/TiO_2_ 30	1.36	16.54	15.18	2.03	6.00	25.21	46.10
NBR/TiO_2_ 70	2.11	18.98	16.87	1.35	5.50	24.10	58.40
NBR/TiO_2_ 90	3.00	22.20	19.20	1.47	5.90	22.56	64.30
NBR/TiO_2_ 110	1.91	16.40	14.49	1.35	5.57	23.72	70.20

**Table 3:** Mechanical properties of TiO_2_-filled NBR composites with various TiO_2_ loadings.

Sample	σ_b_ (MPa)	ε_b_ (%)	M100 (MPa)	M300 (MPa)	Reinforcing Index	Hardness (Shore A)
Pure NBR	2.82 ± 0.11	550 ± 15	0.71 ± 0.01	1.20 ± 0.04	1.69	35
NBR/TiO_2_ 30	3.77 ± 0.16	565 ± 15	1.01 ± 0.07	2.08 ± 0.05	2.06	45
NBR/TiO_2_ 70	4.16 ± 0.18	621 ± 14	1.21 ± 0.02	2.59 ± 0.05	2.14	48
NBR/TiO_2_ 90	5.68 ± 0.13	768 ± 11	1.45 ± 0.01	2.79 ± 0.04	1.92	49
NBR/TiO_2_ 110	5.24 ± 0.18	699 ± 19	1.50 ± 0.03	2.87 ± 0.03	1.91	51

## Data Availability

Data presented in this study are available on request from the corresponding author.
